# Integrated lncRNA and mRNA Transcriptome Analysis Reveals Candidate Regulatory Networks Associated with Postnatal *Longissimus Dorsi* Muscle Development in Hanper Sheep

**DOI:** 10.3390/biology15191694

**Published:** 2026-09-23

**Authors:** Xiaoyong Chen, Zhen Wang, Rui Yuan, Ying Bai, Lei Liu, Guanghui Li

**Affiliations:** 1 College of Animal Science and Technology, Hebei Agricultural University, Baoding 071000, China; 2 Shenzhen Branch, Guangdong Laboratory of Lingnan Modern Agriculture, Key Laboratory of Livestock and Poultry Multi-Omics of MARA, Agricultural Genomics Institute at Shenzhen, Chinese Academy of Agricultural Sciences, Shenzhen 518124, China; 3 College of Life Science and Food Engineering, Hebei University of Engineering, Handan 056000, China; 4 College of Animal Science and Veterinary Medicine, Huazhong Agricultural University, Wuhan 430070, China

**Keywords:** Hanper sheep, *Longissimus Dorsi* muscle, skeletal muscle development, lncRNA-mRNA co-expression analysis, postnatal muscle development, meat production traits

## Abstract

Skeletal muscle development is a key determinant of meat production and meat-quality traits in sheep, but the coordinated long non-coding RNA (lncRNA) and messenger RNA (mRNA) co-expression landscape underlying postnatal muscle development in Hanper sheep remains unclear. In this study, RNA sequencing was performed on *Longissimus Dorsi* muscle samples from Hanper sheep at 1 month (M1), 7 months (M7), and 13 months (M13) of age to characterize lncRNA and mRNA expression profiles during postnatal growth. A total of 13,940 candidate lncRNAs were identified, and these lncRNAs exhibited typical structural features, including shorter transcript lengths, fewer exons, and shorter open reading frames than mRNAs. Differential expression analysis identified 39, 11, and 174 differentially expressed lncRNAs in M7_vs_M1, M13_vs_M7, and M13_vs_M1, respectively, while 357, 160, and 446 differentially expressed mRNAs were detected in the corresponding comparisons. Functional enrichment analyses showed that differentially expressed mRNAs and co-expressed mRNAs associated with differentially expressed lncRNAs were mainly involved in RNA metabolism, translation, ribosome biogenesis, amino acid biosynthesis, energy and lipid metabolism, extracellular matrix remodeling, and cell adhesion. Several muscle-development-related signaling pathways, including AMPK, insulin, PI3K-Akt, PPAR, mTOR, FoxO, ECM–receptor interaction, and focal adhesion pathways, were also enriched. Integrated lncRNA–mRNA analysis further identified candidate lncRNA-associated differentially expressed genes, including *IGF2*, *FOS*, *ACTC1*, *ADIPOQ*, *SPP1*, *PECAM1*, and *SLC16A7*. These findings provide a comprehensive lncRNA and mRNA transcriptomic resource and reveal candidate regulatory molecules potentially involved in postnatal *Longissimus Dorsi* muscle development in Hanper sheep.

## Simple Summary

Skeletal muscle growth strongly influences meat production and meat quality in sheep, but the molecular changes that occur during postnatal muscle development are not fully understood. In this study, we examined the gene activity of *Longissimus Dorsi* muscle in Hanper sheep at 1, 7, and 13 months of age using RNA sequencing. We focused on both protein-coding genes and long non-coding RNAs, which are RNA molecules that do not produce proteins but may be involved in regulating gene activity. Clear age-related changes were observed in both types of RNA, with the largest differences occurring between 1 and 13 months of age. The associated genes and biological processes were mainly related to protein production, energy and fat metabolism, growth signaling, cell adhesion, and remodeling of muscle structure. Several candidate genes and long non-coding RNA-associated networks were also identified for future study. These findings improve our understanding of how sheep muscle develops after birth and provide useful molecular information for future research on muscle growth and meat-production traits in Hanper sheep.

## 1. Introduction

Skeletal muscle is the major component of edible meat and provides an important source of high-quality animal protein for human consumption [1]. In meat-producing animals, skeletal muscle growth is directly associated with carcass yield, lean meat percentage, and meat-quality traits, including tenderness, juiciness, and water-holding capacity [2]. Muscle development is a complex biological process involving myoblast proliferation and differentiation, muscle fiber formation, postnatal fiber hypertrophy, extracellular matrix remodeling, and metabolic maturation [3]. Because the number, size, and type composition of muscle fibers are closely related to meat-production efficiency and quality attributes, elucidating the molecular mechanisms underlying skeletal muscle development is important for genetic improvement and precision breeding in livestock [4]. In sheep, transcriptomic studies of *Longissimus Dorsi* muscle have provided valuable insights into genes and pathways associated with muscle growth, meat yield, and meat quality, but the regulatory networks controlling postnatal muscle development remain incompletely understood [5].

Hanper sheep is a meat-type sheep population developed from Dorper sheep and Small-tailed Han sheep, combining the favorable growth and meat-production characteristics of Dorper sheep with the adaptability and reproductive performance of Small-tailed Han sheep [6]. Crossbreeding with Dorper rams has been widely used to improve the body weight, carcass weight, meat yield, and intramuscular fat deposition of Small-tailed Han sheep and related local sheep populations [7]. Multi-omics studies of Dorper × Small-tailed Han crossbred sheep have also suggested that epigenetic and transcriptomic differences in the *Longissimus Dorsi* muscle are associated with growth, carcass traits [8], and meat-quality variation [9]. The *Longissimus Dorsi* muscle is commonly used as a representative tissue for evaluating skeletal muscle growth and meat quality in sheep because it contributes substantially to carcass value and reflects changes in muscle fiber characteristics, metabolic status, and meat-quality-related traits [4]. In Hanper sheep, the developmental stages of 1, 7, and 13 months represent distinct phases of postnatal muscle growth, from early postnatal development to rapid growth and later maturation. Previous phenotypic and miRNA profiling of Hanper sheep *Longissimus Dorsi* muscle across these stages provided an initial basis for understanding postnatal muscle development; however, the coordinated mRNA and lncRNA regulatory landscape in this breed remains largely unknown.

Messenger RNAs represent the protein-coding component of the transcriptome and provide a direct molecular layer for identifying genes involved in skeletal muscle growth and functional maturation [10,11]. With the development of high-throughput RNA sequencing, transcriptome profiling has been widely used to identify differentially expressed genes and biological pathways associated with economically important traits in livestock [12,13]. In skeletal muscle, changes in mRNA expression are closely related to myogenesis, muscle fiber hypertrophy [14], extracellular matrix organization [8], mitochondrial function, energy metabolism [15], lipid deposition, and intracellular signaling pathways. Previous transcriptomic studies in sheep *Longissimus Dorsi* or *Longissimus thoracis* muscle have identified candidate genes and pathways associated with growth performance, muscle development, carcass traits [4], meat tenderness, intramuscular fat deposition, and other meat-quality traits [16]. However, most mRNA-centered studies have mainly focused on protein-coding genes or individual biological traits, providing limited information on how coding genes are coordinated with non-coding regulatory molecules during postnatal muscle development.

Long non-coding RNAs (lncRNAs) are a major class of non-coding transcripts that are generally longer than 200 nucleotides and lack evident protein-coding potential [17]. Increasing evidence indicates that lncRNAs are not merely transcriptional by-products, but important regulatory molecules involved in gene expression regulation at epigenetic, transcriptional, post-transcriptional, and translational levels [18]. In skeletal muscle, lncRNAs have been reported to participate in myoblast proliferation, cell-cycle withdrawal, myogenic differentiation, muscle fiber formation, muscle regeneration, and fiber-type specification. For example, linc-MD1 regulates muscle differentiation by acting as a competing endogenous RNA [19], LncMyoD promotes terminal myogenic differentiation by modulating mRNA translation [20], and Linc-RAM enhances myogenic differentiation through interaction with MyoD [21]. In livestock, increasing studies have also identified lncRNAs associated with skeletal muscle growth, meat production [22], and muscle-related economic traits [23], suggesting that lncRNAs may represent an important regulatory layer in animal muscle development [24]. Recent studies have further highlighted the importance of lncRNA-associated regulatory networks in farm-animal skeletal muscle. For example, lncRNAs have been summarized as important regulators of myogenesis and lipogenesis in pigs, cattle, sheep, and poultry, and functional studies in sheep have shown that lncRNA GTL2 regulates myoblast proliferation and differentiation via the PKA-CREB pathway, while the LOC121818100/Novel-miR-400/SSRP1 ceRNA axis contributes to sheep skeletal muscle growth and injury repair [25,26,27]. In sheep, genome-wide lncRNA profiling and lncRNA-mRNA co-expression analyses have revealed candidate lncRNAs and target genes potentially involved in muscle growth, metabolism, cell proliferation, apoptosis, and muscle fiber formation [28]. Nevertheless, compared with mRNAs, the functional annotation of lncRNAs remains less complete because many lncRNAs show species-, tissue-, and developmental-stage-specific expression patterns. In addition, many lncRNA-associated mRNAs are inferred from genomic proximity or expression correlation, and therefore integrated lncRNA-mRNA analyses require cautious interpretation and further biological validation.

Although previous mRNA-, miRNA-, and lncRNA-sequencing studies have advanced our understanding of skeletal muscle development in sheep and other livestock species, the integrated mRNA and lncRNA expression landscape associated with postnatal *Longissimus Dorsi* muscle development in Hanper sheep remains insufficiently characterized. In particular, it remains unclear which stage-associated coding genes, biological pathways, and lncRNA-associated co-expression networks contribute to postnatal muscle growth and maturation in this breed. Compared with previous investigations that mainly focused on single transcriptomic layers, individual developmental stages, or other breeds and livestock species, the present study was designed to integrate mRNA and lncRNA transcriptome profiles from the same Hanper sheep *Longissimus Dorsi* muscle samples across three representative postnatal developmental stages. Specifically, *Longissimus Dorsi* muscle samples from Hanper sheep at 1, 7, and 13 months of age were subjected to RNA sequencing, followed by differential expression analysis, functional enrichment analysis, cis-target prediction, lncRNA-mRNA co-expression analysis, and protein–protein interaction network construction. This study aimed to identify candidate genes, lncRNAs, biological pathways, and lncRNA-associated co-expression networks potentially involved in postnatal skeletal muscle development, thereby providing a transcriptomic basis for understanding muscle growth regulation and meat-production traits in Hanper sheep.

## 2. Materials and Methods

### 2.1. Animals and Longissimus Dorsi Muscle Collection

The animal experiment and sample collection procedures were performed as previously described [6]. Briefly, a total of nine healthy uncastrated Hanper rams were selected from Hebei Liansheng Agricultural Development Co., Ltd. (Zhuozhou, China). The animals were divided into three postnatal developmental stages according to age: 1 month old (M1), 7 months old (M7), and 13 months old (M13), with three biological replicates in each group. Within each developmental stage, animals with similar body weights and no genetic kinship were selected to minimize individual variation. All animals were raised at the same farm under a standardized management system. No specific experimental feeding treatment or dietary intervention was applied in this study, and animals at different developmental stages were fed according to the routine age-appropriate feeding practices of the farm.

All animal procedures were approved by the Animal Welfare and Ethics Committee of Hebei Agricultural University, and were conducted in accordance with relevant animal welfare guidelines and regulations. The approval number was 2020070. Before slaughter, the animals were subjected to routine health inspection and handled under appropriate welfare conditions. The sheep were humanely slaughtered according to the “Livestock and Poultry Slaughtering Operation Regulations for Sheep” (NY/T 3469−2019). Immediately after slaughter, *Longissimus Dorsi* muscle samples were collected, rapidly frozen in liquid nitrogen, and then stored at −80 °C until RNA extraction.

### 2.2. RNA Extraction, Library Construction, and Sequencing

Total RNA was extracted from *Longissimus Dorsi* muscle samples using TRIzol reagent (Invitrogen, Carlsbad, CA, USA) according to the manufacturer’s instructions. RNA degradation and contamination were evaluated using 1% agarose gel electrophoresis. RNA purity was assessed using a NanoPhotometer^®^ spectrophotometer (IMPLEN, Westlake Village, CA, USA), and RNA concentration was measured using the Qubit^®^ RNA Assay Kit with a Qubit^®^ 2.0 Fluorometer (Life Technologies, Carlsbad, CA, USA). RNA integrity was further evaluated using the RNA Nano 6000 Assay Kit on an Agilent Bioanalyzer 2100 system (Agilent Technologies, Santa Clara, CA, USA). Only RNA samples that passed quality control were used for subsequent library construction.

For lncRNA and mRNA transcriptome profiling, sequencing libraries were constructed from qualified total RNA samples. After library quality assessment, the prepared libraries were sequenced on an Illumina platform, generating 150 bp paired-end reads for nine *Longissimus Dorsi* muscle samples, including three biological replicates each from the M1, M7, and M13 groups. The sequencing depth and data quality metrics for each sample are provided in Table S1. The obtained sequencing data were subsequently used for quality control, reference genome alignment, transcriptome assembly, lncRNA identification, expression quantification, differential expression analysis, and functional annotation.

### 2.3. Sequencing Data Quality Control, Read Alignment, and Transcriptome Assembly

Raw sequencing reads were first subjected to quality filtering to remove low-quality reads and obtain clean reads for downstream bioinformatics analyses. For each sample, sequencing quality was evaluated based on raw reads, clean reads, clean bases, sequencing error rate, Q20, Q30, and GC content. Across the nine libraries, 94,633,718–118,212,162 raw reads and 91,319,600–114,941,852 clean reads were obtained, corresponding to 13.70–17.24 Gb clean bases per sample. The sequencing error rate was 0.01%, Q20 values ranged from 97.45% to 97.97%, Q30 values ranged from 93.31% to 94.72%, and GC content ranged from 53.66% to 55.99% (Table S1). All subsequent analyses were performed using clean reads that passed quality control.

The clean reads were aligned to the sheep reference genome Ovis aries Oar_v4.0 and its corresponding genome annotation using HISAT2 v2.1.0. Mapping statistics, including total mapped reads, multiple mapped reads, uniquely mapped reads, strand mapping information, non-splice reads, splice reads, and properly paired reads, were calculated to assess the quality of reference genome alignment. The total mapped rate ranged from 86.22% to 93.31%, and the uniquely mapped rate ranged from 69.33% to 78.01% among the nine samples (Table S1). The resulting alignment files were then used for transcriptome assembly. StringTie v1.3.4 was applied to assemble transcripts based on the HISAT2 alignment results and to generate transcript structural information for downstream mRNA expression analysis and lncRNA identification. Transcript expression levels were quantified using StringTie v1.3.4. For each annotated mRNA, lncRNA, and transcript of uncertain coding potential (TUCP), both raw read counts and fragments per kilobase of transcript per million mapped fragments (FPKM) values were obtained. Raw read counts were used as the input for differential expression analysis, whereas FPKM values were used to represent transcript expression abundance and for expression-based visualization analyses, including sample correlation analysis and PCA. To assess the consistency among biological replicates, sample-to-sample expression correlations were calculated based on transcript expression levels using Pearson correlation coefficients. Principal component analysis (PCA) was further performed using the log2(FPKM + 1)-transformed expression matrix of expressed mRNAs and lncRNAs to evaluate the global transcriptomic similarity among biological replicates and developmental stages.

### 2.4. Identification, Characterization, and Differential Expression Analysis of lncRNAs

Candidate lncRNAs were identified from the assembled transcriptome using a stepwise filtering strategy. First, transcripts from all samples were merged using Cuffmerge in the Cufflinks package v2.2.1, and transcripts with uncertain strand information were removed. The merged transcripts were then filtered according to transcript structure, transcript length, annotation information, expression abundance, and coding potential. Transcripts with fewer than two exons or a length of no more than 200 bp were removed. Cuffcompare in the Cufflinks package v2.2.1 was used to compare the assembled transcripts with the reference genome annotation, and transcripts overlapping annotated protein-coding exons were excluded. Transcripts with low expression abundance were further removed using an FPKM threshold of 0.5. Coding potential was predicted using CPC2 v1.0.1, CNCI v2.0, and PfamScan v1.6 against the Pfam protein family database, and only transcripts consistently predicted as non-coding by all three approaches were retained as candidate lncRNAs.

The identified lncRNAs were classified according to their genomic locations, including long intergenic non-coding RNAs (lincRNAs), antisense lncRNAs, and intronic lncRNAs. To characterize the structural features of lncRNAs, transcript length, exon number, and open reading frame (ORF) length were compared among annotated lncRNAs, novel lncRNAs, and mRNAs. Differential expression analysis of lncRNAs was performed using edgeR v3.22.5 based on the raw read count matrix. Pairwise comparisons were conducted among the three developmental stages, including M7_vs_M1, M13_vs_M7, and M13_vs_M1. LncRNAs with a q-value < 0.05 and |log2FC| ≥ 1 were considered differentially expressed lncRNAs.

### 2.5. Cis-Target Prediction, Co-Expression Analysis, and Functional Enrichment Analysis of mRNAs Associated with Differentially Expressed lncRNAs

To explore the potential functions of differentially expressed lncRNAs, both cis-target prediction and lncRNA-mRNA co-expression analysis were performed. For cis-target prediction, protein-coding genes located within 100 kb upstream or downstream of each lncRNA locus, as well as protein-coding genes overlapping with lncRNA loci on the sense or antisense strand, were identified as candidate cis-associated mRNAs. The candidate cis-associated lncRNA-mRNA pairs identified by genomic co-location analysis are provided in Table S2. In parallel, lncRNA-mRNA co-expression analysis was performed based on expression correlations between differentially expressed lncRNAs and mRNAs across the nine *Longissimus Dorsi* muscle samples. For each developmental comparison, including M7_vs_M1, M13_vs_M7, and M13_vs_M1, differentially expressed lncRNAs were used as the input lncRNA set. Pearson correlation analysis was performed between the expression levels of differentially expressed lncRNAs and mRNAs, and mRNAs with an absolute Pearson correlation coefficient greater than 0.95 were retained as lncRNA-associated co-expressed mRNAs. These predicted associations were used only to infer potential functional relationships and were not considered direct evidence of lncRNA-mediated regulation.

Functional enrichment analysis was then performed to investigate the biological functions and pathways associated with the lncRNA-associated co-expressed mRNAs. Gene Ontology (GO) enrichment analysis was conducted against the Gene Ontology database using GOseq v1.32.0, which corrects for gene length bias based on the Wallenius non-central hypergeometric distribution. GO terms were classified into three categories: biological process, cellular component, and molecular function. Kyoto Encyclopedia of Genes and Genomes (KEGG) pathway enrichment analysis was performed against the KEGG database using KOBAS v2.0 based on the hypergeometric test. Enriched GO terms and KEGG pathways were used to infer the potential biological roles associated with differentially expressed lncRNAs during postnatal *Longissimus Dorsi* muscle development in Hanper sheep.

### 2.6. Differential Expression and Functional Enrichment Analysis of mRNAs

Differential expression analysis of mRNAs was performed using edgeR v3.22.5 based on the raw read count matrix. Pairwise comparisons were conducted among the three postnatal developmental stages, including M7_vs_M1, M13_vs_M7, and M13_vs_M1. Transcripts annotated as mRNAs were extracted for mRNA-level differential expression analysis. In each comparison, mRNAs with a q-value < 0.05 and |log2FC| ≥ 1 were considered differentially expressed mRNAs.

To investigate the biological functions of differentially expressed mRNAs, GO and KEGG enrichment analyses were performed for each developmental comparison. GO enrichment analysis was conducted using GOseq v1.32.0. GO terms were classified into biological process, cellular component, and molecular function categories. KEGG pathway enrichment analysis was performed using KOBAS v2.0 based on the hypergeometric test. Enriched GO terms and KEGG pathways were used to identify biological processes and signaling pathways potentially associated with postnatal *Longissimus Dorsi* muscle development in Hanper sheep.

### 2.7. Integrated lncRNA-mRNA Co-Expression Analysis and Protein–Protein Interaction Network Construction

To identify candidate mRNAs potentially associated with differentially expressed lncRNAs during postnatal muscle development, an integrated lncRNA-mRNA co-expression analysis was performed. For each developmental comparison, including M7_vs_M1, M13_vs_M7, and M13_vs_M1, upregulated mRNAs, downregulated mRNAs, mRNAs co-expressed with upregulated lncRNAs, and mRNAs co-expressed with downregulated lncRNAs were compared using Venn analysis. The overlapping genes between differentially expressed mRNAs and lncRNA-associated co-expressed mRNAs were defined as candidate lncRNA-associated differentially expressed mRNAs.

Protein–protein interaction (PPI) networks were constructed for the overlapping protein-coding genes to further evaluate their potential functional associations. The PPI analysis was performed using the STRING database v10.5. For genes without direct interaction records in sheep, homolog-based mapping was applied to identify corresponding protein interaction information. In the PPI networks, nodes represented proteins encoded by the overlapping genes, and edges represented predicted protein associations supported by different evidence sources in STRING.

## 3. Results

### 3.1. Differential mRNA Expression Profiles During Postnatal Muscle Development

To characterize the transcriptional changes associated with postnatal muscle development in Hanper sheep, differentially expressed mRNAs, hereafter referred to as differentially expressed genes (DEGs), were identified among the three developmental comparisons: M7_vs_M1, M13_vs_M7, and M13_vs_M1. A summary of RNA-seq data quality and mapping statistics is provided in Table S1. Across the nine libraries, 91.32–114.94 million clean reads were obtained per sample, with Q30 values ranging from 93.31% to 94.72% and GC content ranging from 53.66% to 55.99%. The total mapped rate ranged from 86.22% to 93.31%, and the uniquely mapped rate ranged from 69.33% to 78.01%, indicating that the sequencing data were of sufficient quality for downstream transcriptome analyses. Before differential expression analysis, PCA was performed to evaluate the overall consistency of the RNA-seq samples. The PCA plot showed that the samples generally clustered according to developmental stage, with M1 samples clearly separated from M7 and M13 samples along PC1, and M7 and M13 samples mainly separated along PC2 (Figure S1). These results indicated acceptable reproducibility among biological replicates and developmental-stage-associated transcriptomic differences. As shown in Figure 1A, the largest number of DEGs was detected in the M13_vs_M1 comparison, with a total of 446 DEGs, including 266 upregulated and 180 downregulated mRNAs. In the M7_vs_M1 comparison, 357 DEGs were identified, of which 220 were upregulated and 137 were downregulated. By contrast, the M13_vs_M7 comparison showed fewer transcriptional changes, with 160 DEGs, including 86 upregulated and 74 downregulated mRNAs. These results indicate that the overall mRNA expression profile changed more markedly across the full developmental interval from 1 to 13 months than during the later transition from 7 to 13 months.

Hierarchical clustering analysis showed that the differentially expressed mRNAs exhibited stage-associated expression patterns across the nine *Longissimus Dorsi* muscle samples (Figure 1B). Samples from the same developmental stage generally clustered together, suggesting good biological consistency within groups and clear transcriptional separation among developmental stages. The Venn diagram showed that 169, 79, and 263 DEGs were specifically detected in the M7_vs_M1, M13_vs_M7, and M13_vs_M1 comparisons, respectively. In addition, 43 DEGs were shared between M7_vs_M1 and M13_vs_M7, 145 DEGs were shared between M7_vs_M1 and M13_vs_M1, and 38 DEGs were shared between M13_vs_M7 and M13_vs_M1; however, no DEG was common to all three comparisons (Figure 1C). Volcano plots further illustrated the distribution of upregulated and downregulated mRNAs in each comparison, consistent with the DEG counts shown in Figure 1A,D). Together, these results suggest that mRNA expression changes in Hanper sheep *Longissimus Dorsi* muscle are developmentally regulated, with the most pronounced transcriptional divergence occurring between M1 and M13.

### 3.2. Functional Enrichment Analysis of Differentially Expressed mRNAs

To further explore the potential biological functions of the DEGs identified during postnatal muscle development, GO and KEGG enrichment analyses were performed for the three developmental comparisons. In the M7_vs_M1 comparison, GO enrichment analysis showed that DEGs were mainly associated with RNA metabolic process, nucleobase-containing compound metabolic and biosynthetic processes, mitochondrial outer membrane, organelle outer membrane, mitochondrial envelope, nucleic acid binding, kinase regulator activity, and sequence-specific DNA binding (Figure 2A). KEGG pathway analysis showed that these DEGs were enriched in several signaling and metabolic pathways, including adipocytokine signaling pathway, AMPK signaling pathway, glycine, serine and threonine metabolism, RNA degradation, Toll-like receptor signaling pathway, and type II diabetes mellitus (Figure 2B).

In the M13_vs_M1 comparison, enriched GO terms were mainly related to RNA metabolic process, cellular response to chemical stimulus, regulation of macromolecule metabolic process, coated membrane, membrane coat, nucleus, binding, DNA binding, and sequence-specific DNA binding (Figure 2C). KEGG enrichment analysis indicated that DEGs in M13_vs_M1 were involved in pathways such as spliceosome, TNF signaling pathway, cell adhesion molecules, cAMP signaling pathway, estrogen signaling pathway, glycine, serine and threonine metabolism, leukocyte transendothelial migration, and antigen processing and presentation (Figure 2D).

In the M13_vs_M7 comparison, GO enrichment analysis revealed that DEGs were mainly associated with cellular membrane organization, membrane organization, intracellular protein transport, cell, cell part, intracellular component, transferase activity, zinc ion binding, and ion binding (Figure 2E). KEGG pathways enriched in this comparison included calcium signaling pathway, apoptosis, NF-kappa B signaling pathway, phosphatidylinositol signaling system, protein processing in endoplasmic reticulum, ribosome biogenesis in eukaryotes, tight junction, and glycosaminoglycan biosynthesis (Figure 2F). Overall, these enrichment results suggest that the transcriptional changes during Hanper sheep *Longissimus Dorsi* muscle development were closely associated with RNA metabolism, membrane and vesicle organization, signal transduction, metabolic regulation, and cellular structural remodeling.

### 3.3. Identification and Characterization of lncRNAs in Hanper Sheep Longissimus Dorsi Muscle

To identify lncRNAs expressed in the *Longissimus Dorsi* muscle of Hanper sheep, transcriptome assembly and a stringent stepwise filtering strategy were performed. A total of 286,061 assembled transcripts were initially obtained. After filtering based on transcript structure, transcript length, annotation information, expression abundance, and coding potential, 13,940 candidate lncRNA transcripts were retained for downstream analysis (Figure 3A). Coding-potential prediction was further performed using CPC2, CNCI, and PFAM. The intersection of these three approaches contained 13,940 transcripts, which were consistently classified as non-coding and were therefore defined as the final candidate lncRNA set (Figure 3B).

The identified lncRNAs were classified according to their genomic locations. Intronic lncRNAs accounted for the largest proportion of the candidate lncRNAs, representing 66.9% of the total, followed by lincRNAs and antisense lncRNAs, which accounted for 25.9% and 7.2%, respectively (Figure 3C). The structural characteristics of lncRNAs were then compared with those of mRNAs. Both annotated and novel lncRNAs showed shorter transcript lengths, fewer exons, and shorter open reading frames (ORFs) than mRNAs (Figure 3D). These structural features were consistent with the typical characteristics of lncRNAs, supporting the reliability of the lncRNA identification and providing a basis for subsequent differential expression and target prediction analyses.

### 3.4. Differential Expression Profiles of lncRNAs During Postnatal Muscle Development

To investigate the potential regulatory roles of lncRNAs during postnatal muscle development, differentially expressed lncRNAs were identified among the three developmental comparisons. As shown in Figure 4A, the M13_vs_M1 comparison exhibited the largest number of differentially expressed lncRNAs, with a total of 174 differentially expressed lncRNAs, including 20 upregulated and 154 downregulated lncRNAs. In the M7_vs_M1 comparison, 39 differentially expressed lncRNAs were detected, of which 15 were upregulated and 24 were downregulated. The M13_vs_M7 comparison showed the fewest changes, with 11 differentially expressed lncRNAs, including 5 upregulated and 6 downregulated lncRNAs. These results indicate that lncRNA expression changed most prominently across the full developmental interval from M1 to M13, whereas relatively fewer lncRNAs were altered during the later transition from M7 to M13.

The Venn diagram further showed comparison-specific and shared differentially expressed lncRNAs among the three developmental comparisons (Figure 4B). Specifically, 17, 1, and 152 differentially expressed lncRNAs were unique to M7_vs_M1, M13_vs_M7, and M13_vs_M1, respectively. In addition, 5 differentially expressed lncRNAs were shared between M7_vs_M1 and M13_vs_M7, 17 were shared between M7_vs_M1 and M13_vs_M1, and 5 were shared between M13_vs_M7 and M13_vs_M1. No differentially expressed lncRNA was shared by all three comparisons. Volcano plots showed the overall distribution of upregulated and downregulated lncRNAs in each comparison, further supporting the marked difference in lncRNA expression changes among developmental stages (Figure 4C). Together, these results suggest that lncRNA expression profiles are dynamically regulated during Hanper sheep *Longissimus Dorsi* muscle development, with the most pronounced lncRNA expression divergence occurring between M1 and M13.

### 3.5. Functional Enrichment Analysis of lncRNA-Associated Co-Expressed mRNAs

To provide additional genomic-context information for lncRNA-associated mRNA prediction, cis-target prediction was performed based on genomic co-location analysis. Protein-coding genes located within 100 kb upstream or downstream of lncRNA loci, as well as genes overlapping with lncRNA loci on the sense or antisense strand, were identified as candidate cis-associated mRNAs. In total, 146,680 candidate cis-associated lncRNA-mRNA pairs were identified, involving 13,937 lncRNA transcripts and 16,639 protein-coding gene IDs (Table S2). These cis-associated pairs included upstream, downstream, antisense, and sense-overlapping genomic relationships, providing complementary genomic evidence for interpreting lncRNA-associated mRNA relationships. In the M7_vs_M1 comparison, GO enrichment analysis showed that lncRNA-associated co-expressed mRNAs were mainly associated with ncRNA metabolic process, organic substance biosynthetic process, organic cyclic compound metabolic process, ribonucleoprotein complex, ribosome, cytoplasm, kinase regulator activity, heterocyclic compound binding, and organic cyclic compound binding (Figure 5A). KEGG enrichment analysis indicated that these lncRNA-associated co-expressed mRNAs were involved in adipocytokine signaling pathway, AMPK signaling pathway, insulin signaling pathway, PI3K-Akt signaling pathway, PPAR signaling pathway, ECM–receptor interaction, aminoacyl-tRNA biosynthesis, biosynthesis of amino acids, ribosome, and ribosome biogenesis in eukaryotes (Figure 5B).

In the M13_vs_M1 comparison, enriched GO terms were mainly related to cellular biosynthetic process, organic substance biosynthetic process, biosynthetic process, organelle, intracellular organelle, intracellular component, heterocyclic compound binding, organic cyclic compound binding, and binding (Figure 5C). KEGG analysis showed enrichment of pathways associated with adipocytokine signaling pathway, AMPK signaling pathway, insulin signaling pathway, aminoacyl-tRNA biosynthesis, biosynthesis of amino acids, glycine, serine and threonine metabolism, purine metabolism, pyrimidine metabolism, ribosome, and signaling pathways regulating pluripotency of stem cells (Figure 5D).

In the M13_vs_M7 comparison, GO enrichment analysis showed that lncRNA-associated co-expressed mRNAs were mainly associated with cellular macromolecule biosynthetic process, nuclear transport, nucleocytoplasmic transport, cell, cell part, intracellular component, transition metal ion binding, zinc ion binding, and hydrolase activity acting on carbon–nitrogen bonds (Figure 5E). KEGG pathways enriched in this comparison included ECM–receptor interaction, focal adhesion, FoxO signaling pathway, mTOR signaling pathway, p53 signaling pathway, neurotrophin signaling pathway, adrenergic signaling in cardiomyocytes, and circadian rhythm (Figure 5F). Overall, the functional enrichment of lncRNA-associated co-expressed mRNAs suggested that differentially expressed lncRNAs may be associated with postnatal muscle development through biological processes related to RNA metabolism, protein synthesis, energy and nutrient signaling, extracellular matrix organization, cell adhesion, and intracellular signal transduction.

### 3.6. Integrated Analysis of lncRNA-Associated Co-Expressed mRNAs and DEGs

To further prioritize candidate mRNAs potentially associated with differentially expressed lncRNAs, lncRNA-associated co-expressed mRNAs were intersected with DEGs in each developmental comparison. In the M7_vs_M1 comparison, seven overlapping mRNAs were identified between DEGs and lncRNA-associated co-expressed mRNAs (Figure 6A). These included one upregulated mRNA co-expressed with downregulated lncRNAs, three upregulated mRNAs co-expressed with upregulated lncRNAs, and three downregulated mRNAs co-expressed with downregulated lncRNAs. The corresponding annotated genes were *ABCA1*, *ACTC1*, *BTG2*, *FOS*, *IGF2*, *PSAT1*, and *USP45*. PPI analysis of these genes showed a small interaction network involving several development- and metabolism-related genes, including *IGF2*, *FOS*, *ABCA1*, and *BTG2* (Figure 6D).

In the M13_vs_M1 comparison, 28 overlapping mRNAs were identified between DEGs and lncRNA-associated co-expressed mRNAs (Figure 6B). After gene annotation and PPI mapping, 25 protein-coding genes were included in the PPI network, including *ACTC1*, *ADIPOQ*, *BAZ2A*, *EIF2B3*, *EIF4G1*, *FAM214A*, *FBXL20*, *FOS*, *HBS1L*, *IGF2*, *KMT5A*, *MPP5*, *NRK*, *PDE8A*, *PECAM1*, *PSAT1*, *RBM8A*, *SEPTIN7*, *SLC16A7*, *SNX25*, *SPP1*, *STAU2*, *VEZF1*, *ZNF683*, and *ZNF7*. The PPI network contained genes involved in growth regulation, extracellular matrix and adhesion-related processes, translation regulation, metabolic transport, and signal transduction, such as *IGF2*, *FOS*, *SPP1*, *ADIPOQ*, *PECAM1*, *ACTC1*, *EIF4G1*, and *SLC16A7* (Figure 6E). In contrast, no overlapping mRNA was detected between DEGs and lncRNA-associated co-expressed mRNAs in the M13_vs_M7 comparison (Figure 6C). This absence of overlap may be related to the relatively limited transcriptomic changes during the later developmental transition from M7 to M13, as this comparison showed fewer DEGs and the fewest differentially expressed lncRNAs among the three developmental comparisons. In addition, the integrated analysis used a relatively stringent intersection strategy, requiring candidate mRNAs to be both differentially expressed and associated with differentially expressed lncRNAs. Therefore, the lack of overlapping genes does not indicate an absence of biological changes during the M13_vs_M7 transition, but rather suggests that the mRNA-level changes and lncRNA-associated co-expression changes at this stage may be less coordinated than those observed across the broader M1-to-M13 developmental interval.

## 4. Discussion

Postnatal skeletal muscle development is accompanied by extensive remodeling of both coding and non-coding transcriptomes. In the present study, RNA-seq analysis of *Longissimus Dorsi* muscle from Hanper sheep at 1, 7, and 13 months of age revealed dynamic expression profiles of lncRNAs and mRNAs during postnatal growth. A total of 13,940 candidate lncRNAs were identified, and these lncRNAs showed shorter transcript lengths, fewer exons, and shorter open reading frames than mRNAs, which is consistent with the typical structural characteristics of lncRNAs reported in previous sheep skeletal muscle studies [29]. Differential expression analysis further showed that both mRNAs and lncRNAs displayed clear developmental-stage-dependent expression patterns. The largest number of differentially expressed transcripts was observed in M13_vs_M1, whereas M13_vs_M7 showed the fewest changes, suggesting that transcriptomic remodeling was more pronounced from early postnatal development to later maturation than from 7 to 13 months. Similar stage-dependent expression changes in lncRNAs and mRNAs have also been reported in Hu sheep skeletal muscle and prenatal/postnatal sheep muscle, supporting the view that lncRNAs may participate in the fine regulation of skeletal muscle development in a stage-specific manner [24,29]. Consistent with these studies, the present results showed that both mRNAs and lncRNAs exhibited developmental-stage-associated expression patterns in Hanper sheep. However, unlike studies that focused on fetal-to-postnatal transitions or other sheep breeds, the present study specifically examined postnatal *Longissimus Dorsi* muscle development in Hanper sheep from 1 to 13 months of age. The stronger transcriptomic divergence observed in M13_vs_M1 than in M13_vs_M7 suggests that the transition from early postnatal growth to later maturation involves more extensive remodeling of coding and non-coding transcriptomes, whereas the later transition from 7 to 13 months may represent a relatively more moderate maturation stage. Together with our previous observation that muscle fiber area increased progressively from M1 to M13 in Hanper sheep [6], these findings indicate that coordinated changes in lncRNA and mRNA expression may be closely associated with postnatal *Longissimus Dorsi* muscle growth and maturation in this breed.

Functional enrichment analyses further suggested that both differentially expressed mRNAs and lncRNA-associated co-expressed mRNAs were mainly associated with biological processes and pathways related to muscle growth and maturation. In this study, GO terms related to RNA/ncRNA metabolic processes, biosynthetic and metabolic processes, ribonucleoprotein complex, ribosome, membrane and vesicle organization, nuclear transport, and binding-related molecular functions were enriched in different comparisons. These results indicate that transcriptional regulation, macromolecule biosynthesis, intracellular transport, and protein synthesis-related processes may represent important transcriptomic events during postnatal *Longissimus Dorsi* muscle development in Hanper sheep. Similar enrichment of translation- and metabolism-related genes has been reported in sheep skeletal muscle lncRNA studies, in which lncRNA-mRNA co-expression analyses identified candidate lncRNAs and co-expressed genes associated with muscle development, metabolism, cell proliferation, and apoptosis [22]. In addition, pathways such as AMPK, insulin, PI3K-Akt, PPAR, adipocytokine, mTOR, and FoxO signaling were enriched among differentially expressed mRNAs or lncRNA-associated co-expressed mRNAs in this study. These pathways are closely related to nutrient sensing, energy metabolism, protein turnover, adipogenesis, and muscle fiber growth, and have also been implicated in previous transcriptomic studies of sheep skeletal muscle development and meat-quality formation [4]. Notably, ECM–receptor interaction and focal adhesion were also enriched, especially in comparisons involving later developmental stages, suggesting that extracellular matrix remodeling and cell–matrix communication may contribute to muscle structural maturation. Consistent with this interpretation, transcriptomic studies in Tibetan sheep and other sheep populations have also reported enrichment of ECM–receptor interaction, focal adhesion, AMPK, PI3K-Akt, and PPAR signaling pathways during muscle development or meat-quality variation [4,30]. Therefore, the functional enrichment results indicate that lncRNA-associated mRNA regulation may participate in postnatal muscle development through coordinated effects on protein synthesis, metabolic regulation, growth-related signaling, and extracellular matrix remodeling.

The integrated lncRNA-mRNA analysis further prioritized candidate molecules potentially associated with Hanper sheep muscle development. By intersecting differentially expressed mRNAs with lncRNA-associated co-expressed mRNAs of differentially expressed lncRNAs, seven overlapping genes were identified in M7_vs_M1, including *ABCA1*, *ACTC1*, *BTG2*, *FOS*, *IGF2*, *PSAT1*, and *USP45*, whereas a larger set of overlapping genes was observed in M13_vs_M1. Among these candidates, IGF2 is particularly noteworthy because it is a well-known growth-related gene and has been functionally linked to skeletal muscle growth in sheep. For example, lnc-SEMT was reported to promote sheep muscle development and growth by modulating *IGF2* expression, suggesting that IGF2 may be an important candidate gene in lncRNA-associated muscle developmental processes in sheep [31]. In addition, *FOS*, an immediate early gene and AP-1 family component, has been implicated in early activation events during skeletal muscle regeneration, supporting its possible involvement in myogenic activation and remodeling processes [32]. The presence of *ACTC1* in both M7_vs_M1 and M13_vs_M1 also suggests that genes associated with muscle structural organization and contractile function may be involved in the developmental transition of *Longissimus Dorsi* muscle. Moreover, metabolism-related genes such as *ADIPOQ*, *ABCA1*, and *SLC16A7* may be associated with lipid metabolism, intramuscular fat deposition, and substrate transport, which are important biological processes affecting muscle maturation and meat-quality traits. In Tan sheep, *ADIPOQ* has been reported as a lipid-metabolism-related gene associated with intramuscular fat deposition and fatty acid composition [33]. Genes related to extracellular matrix organization and cell communication, such as *SPP1* and *PECAM1*, may also contribute to tissue remodeling during muscle maturation. These candidate genes partially overlap with functional categories reported in previous sheep and livestock muscle studies, including growth regulation, lipid metabolism, extracellular matrix organization, and muscle structural development, supporting the biological relevance of the present findings. Importantly, by integrating differentially expressed mRNAs, differentially expressed lncRNAs, cis-associated relationships, co-expression patterns, and PPI network information in the same Hanper sheep samples, the present study links previously reported muscle-development-related functional categories with lncRNA-associated co-expression patterns in this breed. Therefore, these results provide a focused set of candidate genes and lncRNA-associated networks for future functional studies on postnatal muscle development and meat-production traits in Hanper sheep.

Taken together, this study provides a systematic lncRNA and mRNA transcriptomic resource for understanding postnatal *Longissimus Dorsi* muscle development in Hanper sheep. Our previous miRNA-based study of Hanper sheep suggested that postnatal muscle development is accompanied by progressive changes in muscle fiber morphology and miRNA-mediated regulatory signals associated with skeletal muscle development [6]. The present study extends these findings from the lncRNA and mRNA perspectives and indicates that multiple layers of transcriptomic regulation may contribute to muscle growth and maturation in this breed. However, several limitations should be acknowledged. Although cis-target prediction and lncRNA–mRNA co-expression analysis provided complementary genomic and expression-level evidence for candidate lncRNA–mRNA associations, these results remain computational predictions and cannot establish direct regulatory interactions. In the present study, key differentially expressed lncRNAs and their associated candidate genes were not validated by RT-qPCR because sufficient remaining RNA samples were unavailable. Second, the PPI networks were based on database prediction and homolog-based mapping, which require further experimental validation. Given that lncRNAs can regulate gene expression through diverse molecular mechanisms at epigenetic, transcriptional, post-transcriptional, and translational levels [17,18], future studies should validate the expression patterns and functions of key lncRNAs and candidate genes using RT-qPCR, loss- and gain-of-function assays, and molecular interaction experiments. In particular, candidate genes such as *IGF2*, *FOS*, *ACTC1*, *ADIPOQ*, *SPP1*, *PECAM1*, and *SLC16A7*, together with their associated lncRNAs, may provide useful targets for further investigation of muscle development and meat-production traits in Hanper sheep.

## 5. Conclusions

In conclusion, this study systematically characterized the integrated mRNA and lncRNA transcriptome profiles of *Longissimus Dorsi* muscle in Hanper sheep at three postnatal developmental stages. A total of 13,940 candidate lncRNAs were identified, and both mRNAs and lncRNAs exhibited developmental-stage-associated expression changes, with the most pronounced transcriptomic divergence observed between M13 and M1. Functional enrichment analyses indicated that differentially expressed mRNAs and lncRNA-associated co-expressed mRNAs were mainly involved in RNA metabolism, translation, energy and lipid metabolism, growth-related signaling, extracellular matrix remodeling, and cell adhesion, suggesting that postnatal muscle maturation is accompanied by coordinated remodeling of metabolic, structural, and regulatory pathways. Integrated cis-target prediction, lncRNA-mRNA co-expression analysis, and PPI network analysis further highlighted candidate genes, including IGF2, FOS, ACTC1, ADIPOQ, SPP1, PECAM1, and SLC16A7, together with lncRNA-associated co-expression networks. These findings provide an integrated transcriptomic resource and a focused set of candidate molecules for future functional validation, thereby contributing to a more comprehensive understanding of the molecular regulation of postnatal muscle development and meat-production traits in Hanper sheep.

## Figures and Tables

**Figure 1:**
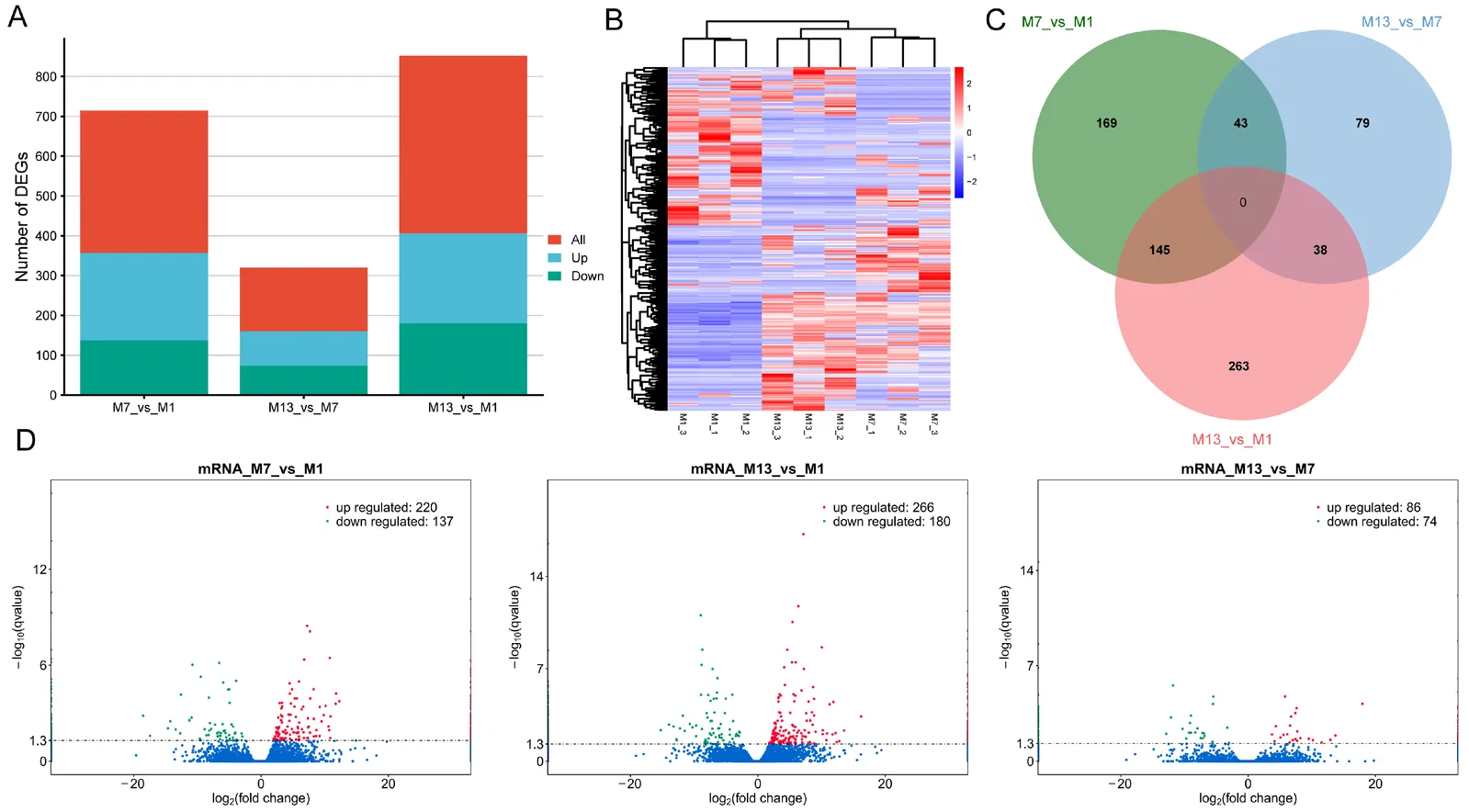
Differential mRNA expression profiles during postnatal *Longissimus Dorsi* muscle development in Hanper sheep. (**A**) Numbers of DEGs in the M7_vs_M1, M13_vs_M7, and M13_vs_M1 comparisons. (**B**) Hierarchical clustering heatmap of differentially expressed mRNAs across the nine *Longissimus Dorsi* muscle samples. (**C**) Venn diagram showing shared and comparison-specific DEGs among the three developmental comparisons. (**D**) Volcano plots showing the distribution of upregulated and downregulated mRNAs in M7_vs_M1, M13_vs_M1, and M13_vs_M7 comparisons. DEGs were defined using q-value < 0.05 and |log2FC| ≥ 1. The horizontal dashed line indicates q-value = 0.05, corresponding to −log10(q-value) = 1.3.

**Figure 2:**
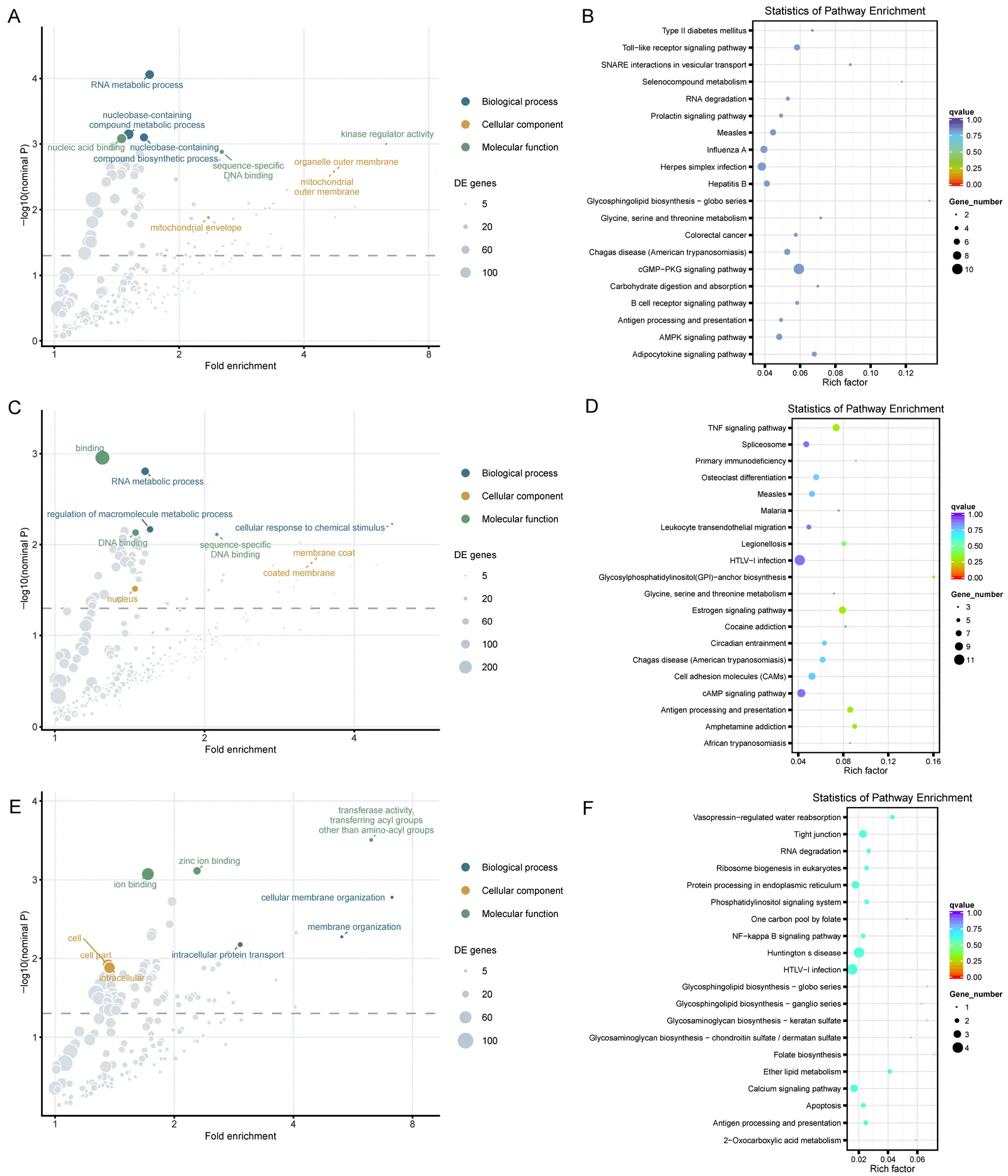
GO and KEGG enrichment analyses of differentially expressed mRNAs during postnatal *Longissimus Dorsi* muscle development in Hanper sheep. (**A**) GO enrichment analysis of DEGs in the M7_vs_M1 comparison. (**B**) KEGG pathway enrichment analysis of DEGs in the M7_vs_M1 comparison. (**C**) GO enrichment analysis of DEGs in the M13_vs_M1 comparison. (**D**) KEGG pathway enrichment analysis of DEGs in the M13_vs_M1 comparison. (**E**) GO enrichment analysis of DEGs in the M13_vs_M7 comparison. (**F**) KEGG pathway enrichment analysis of DEGs in the M13_vs_M7 comparison.

**Figure 3:**
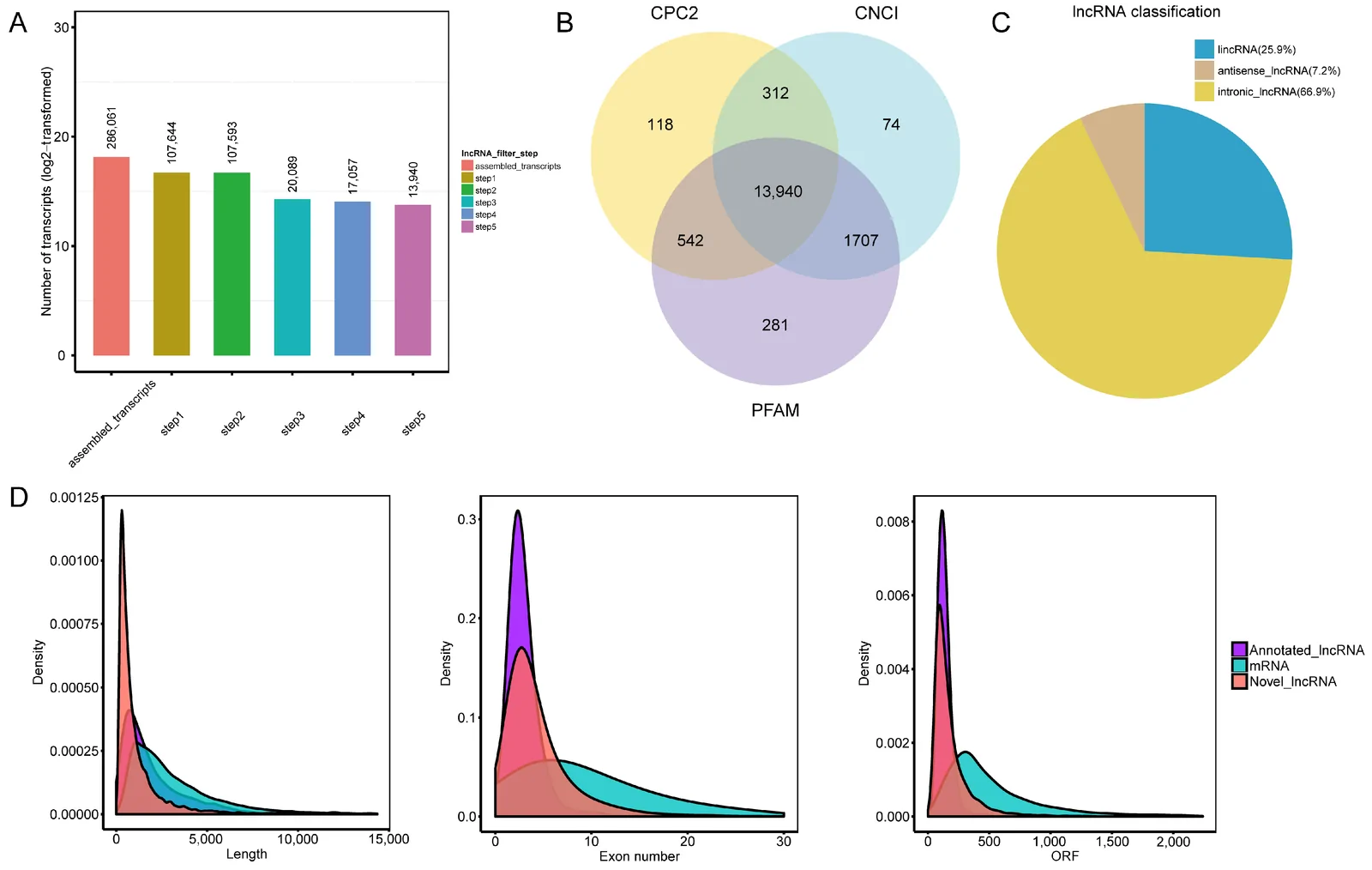
Identification and characterization of lncRNAs in Hanper sheep *Longissimus Dorsi* muscle. (**A**) Stepwise filtering of candidate lncRNA transcripts. A total of 286,061 assembled transcripts were initially obtained, and 13,940 candidate lncRNA transcripts were retained after sequential filtering. (**B**) Venn diagram showing the coding-potential prediction results of CPC2, CNCI, and PFAM. The intersection indicates transcripts consistently predicted as non-coding by all three approaches. (**C**) Classification of identified lncRNAs according to genomic location, including lincRNAs, antisense lncRNAs, and intronic lncRNAs. (**D**) Density distributions of transcript length, exon number, and ORF length among annotated lncRNAs, novel lncRNAs, and mRNAs.

**Figure 4:**
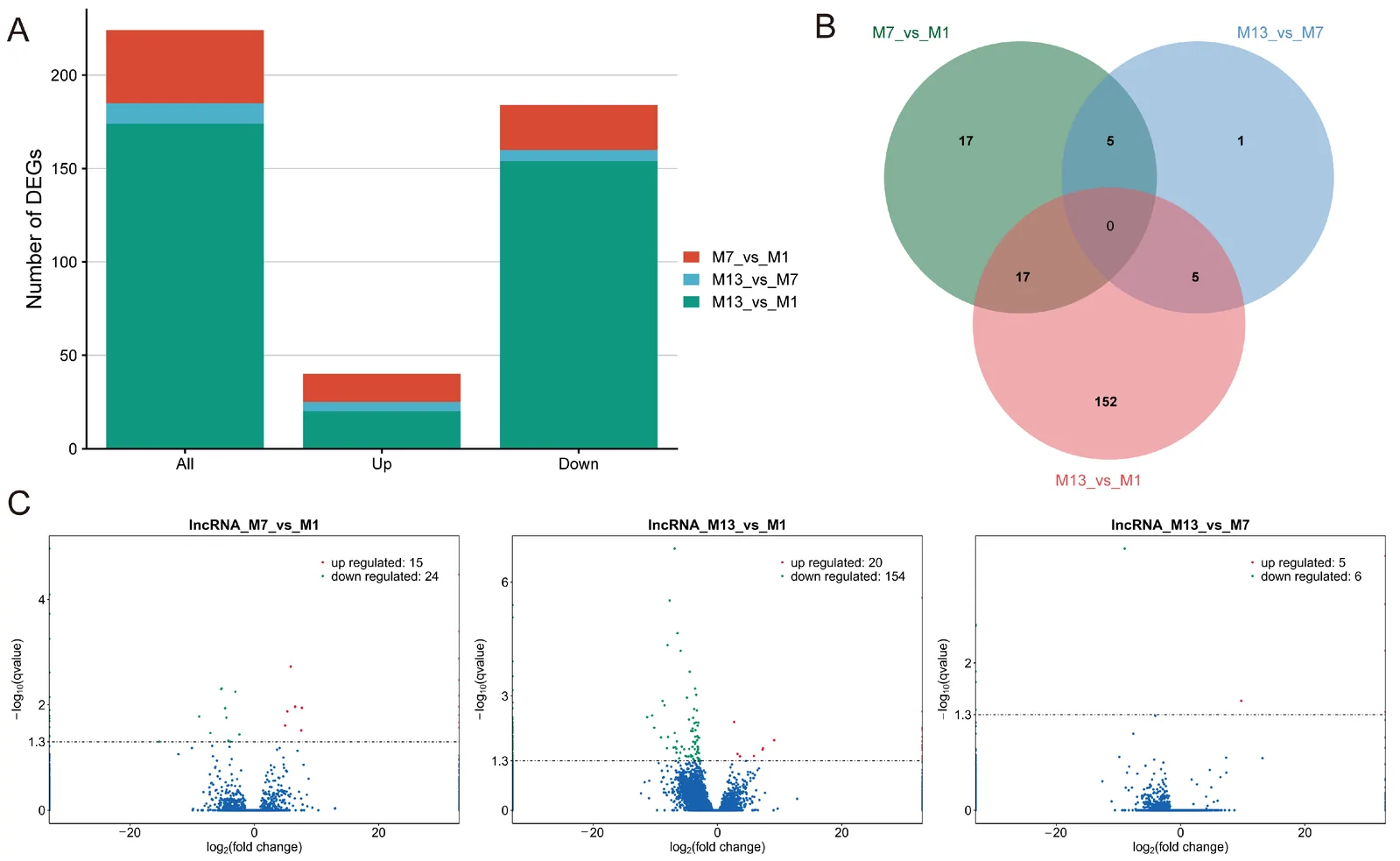
Differential expression profiles of lncRNAs during postnatal *Longissimus Dorsi* muscle development in Hanper sheep. (**A**) Numbers of differentially expressed lncRNAs in the M7_vs_M1, M13_vs_M7, and M13_vs_M1 comparisons. (**B**) Venn diagram showing shared and comparison-specific differentially expressed lncRNAs among the three developmental comparisons. (**C**) Volcano plots showing the distribution of upregulated and downregulated lncRNAs in the M7_vs_M1, M13_vs_M1, and M13_vs_M7 comparisons. Differentially expressed lncRNAs were defined using q-value < 0.05 and |log2FC| ≥ 1. The horizontal dashed line indicates q-value = 0.05, corresponding to −log10(q-value) = 1.3.

**Figure 5:**
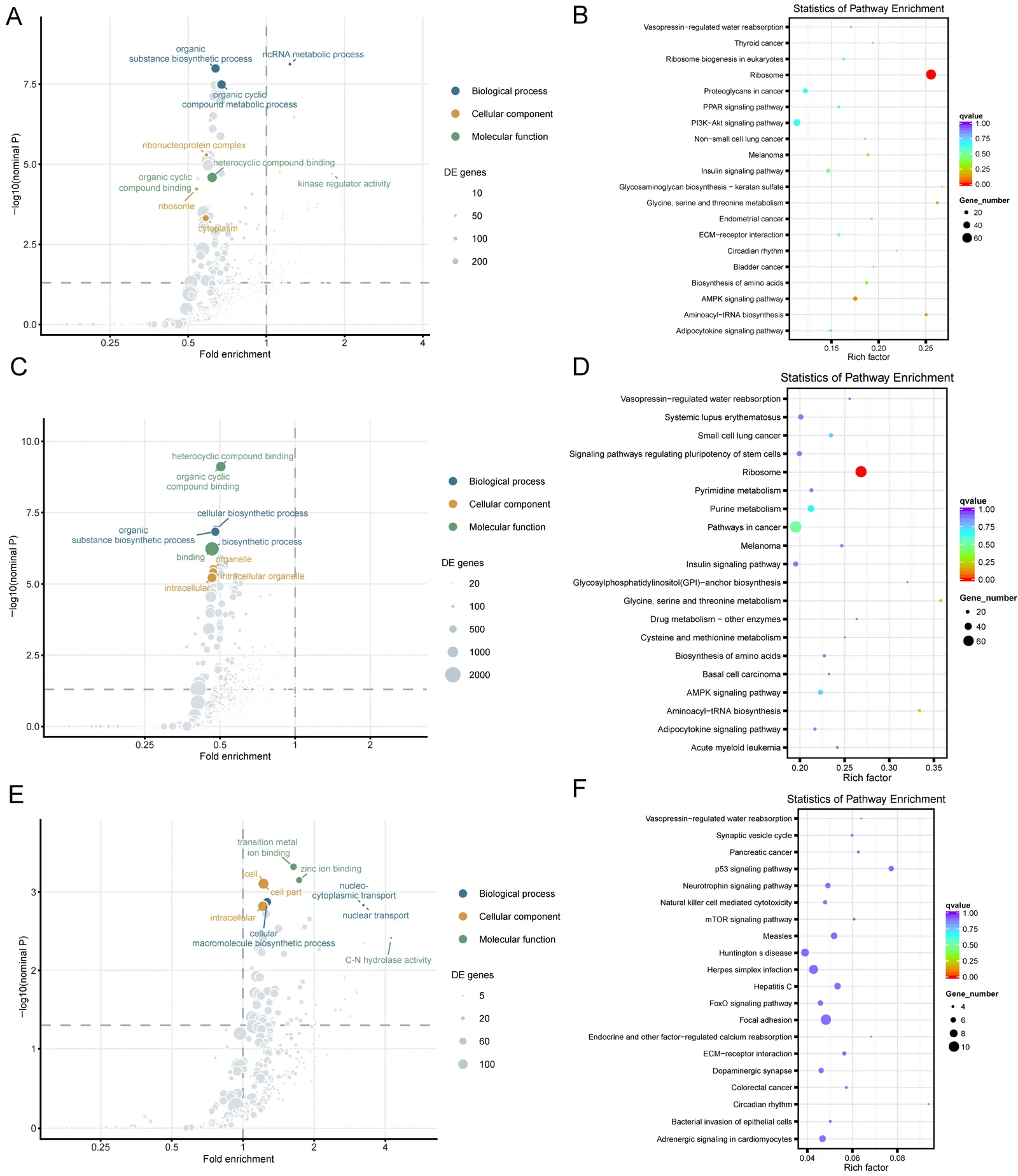
Functional enrichment analysis of lncRNA-associated co-expressed mRNAs during postnatal *Longissimus Dorsi* muscle development in Hanper sheep. (**A**) GO enrichment analysis of lncRNA-associated co-expressed mRNAs in the M7_vs_M1 comparison. (**B**) KEGG pathway enrichment analysis of lncRNA-associated co-expressed mRNAs in the M7_vs_M1 comparison. (**C**) GO enrichment analysis of lncRNA-associated co-expressed mRNAs in the M13_vs_M1 comparison. (**D**) KEGG pathway enrichment analysis of lncRNA-associated co-expressed mRNAs in the M13_vs_M1 comparison. (**E**) GO enrichment analysis of lncRNA-associated co-expressed mRNAs in the M13_vs_M7 comparison. (**F**) KEGG pathway enrichment analysis of lncRNA-associated co-expressed mRNAs in the M13_vs_M7 comparison.

**Figure 6:**
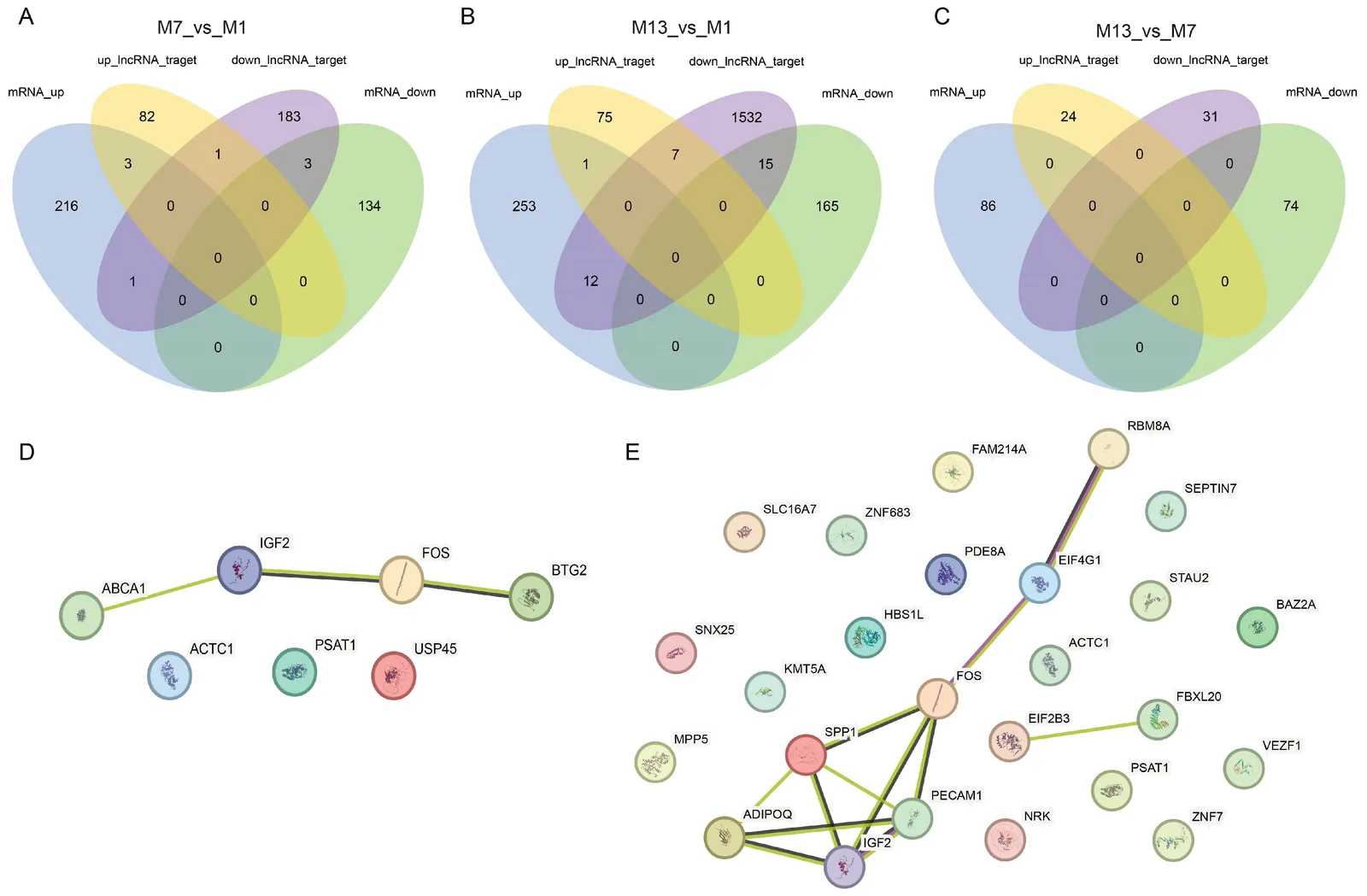
Integrated analysis of differentially expressed mRNAs and lncRNA-associated co-expressed mRNAs during postnatal *Longissimus Dorsi* muscle development in Hanper sheep. (**A**) Venn diagram showing the overlap among upregulated mRNAs, downregulated mRNAs, mRNAs co-expressed with upregulated lncRNAs, and mRNAs co-expressed with downregulated lncRNAs in the M7_vs_M1 comparison. (**B**) Venn diagram showing the overlap among upregulated mRNAs, downregulated mRNAs, mRNAs co-expressed with upregulated lncRNAs, and mRNAs co-expressed with downregulated lncRNAs in the M13_vs_M1 comparison. (**C**) Venn diagram showing the overlap among upregulated mRNAs, downregulated mRNAs, mRNAs co-expressed with upregulated lncRNAs, and mRNAs co-expressed with downregulated lncRNAs in the M13_vs_M7 comparison. (**D**) PPI network of overlapping genes between DEGs and lncRNA-associated co-expressed mRNAs in the M7_vs_M1 comparison. (**E**) PPI network of overlapping genes between DEGs and lncRNA-associated co-expressed mRNAs in the M13_vs_M1 comparison. In the PPI networks, nodes represent proteins encoded by the overlapping genes, and edges represent predicted protein associations. Edge colors indicate different evidence sources supporting the associations in STRING, including curated databases, experimental evidence, gene neighborhood, gene fusion, gene co-occurrence, co-expression, text mining, and protein homology.

## Data Availability

The RNA-seq data generated in this study have been deposited in the NCBI Sequence Read Archive (SRA) under BioProject accession number PRJNA1515387.

## Supplementary Materials

The following supporting information can be downloaded at https://www.mdpi.com/article/10.3390/biology15191694/s1, Figure S1: Principal component analysis (PCA) of RNA-seq samples from Hanper sheep longissimus dorsi muscle; Table S1: Summary of RNA-seq data quality and mapping statistics; Table S2: Co-location analysis of candidate lncRNAs and neighboring protein-coding genes.

## References

[1] National Research Council (US), Committee on Technological Options to Improve the Nutritional Attributes of Animal Products (1988). Designing Foods: Animal Product Options in the Marketplace.

[2] Jan A.T., Lee E.J., Ahmad S., Choi I. (2016). Meeting the Meat: Delineating the Molecular Machinery of Muscle Development. J. Anim. Sci. Technol..

[3] Mohammadabadi M., Bordbar F., Jensen J., Du M., Guo W. (2021). Key Genes Regulating Skeletal Muscle Development and Growth in Farm Animals. Animals.

[4] Wen Y., Li S., Bao G., Wang J., Liu X., Hu J., Zhao F., Zhao Z., Shi B., Luo Y. (2022). Comparative Transcriptome Analysis Reveals the Mechanism Associated With Dynamic Changes in Meat Quality of the Longissimus Thoracis Muscle in Tibetan Sheep at Different Growth Stages. Front. Vet. Sci..

[5] Cheng S., Wang X., Wang Q., Yang L., Shi J., Zhang Q. (2020). Comparative Analysis of *Longissimus Dorsi* Tissue from Two Sheep Groups Identifies Differentially Expressed Genes Related to Growth, Development and Meat Quality. Genomics.

[6] Zhi Y., Hu B., Tian S., Bai Y., Chen X. (2025). Screening and Validation of Key microRNAs Regulating Muscle Development in Hanper Sheep. PLoS ONE.

[7] Xiao C., Liu Y., Zhao W., Liang Y., Cui C., Yang S., Fang W., Miao L., Yuan Z., Lin Z. (2024). The Comparison of Meat Yield, Quality, and Flavor between Small-Tailed Han Sheep and Two Crossbred Sheep and the Verification of Related Candidate Genes. Front. Nutr..

[8] Cheng S., Wang X., Zhang Q., He Y., Zhang X., Yang L., Shi J. (2020). Comparative Transcriptome Analysis Identifying the Different Molecular Genetic Markers Related to Production Performance and Meat Quality in Longissimus Dorsi Tissues of MG × STH and STH Sheep. Genes.

[9] Cao Y., Jin H.-G., Ma H.-H., Zhao Z.-H. (2017). Comparative Analysis on Genome-Wide DNA Methylation in Longissimus Dorsi Muscle between Small Tailed Han and Dorper×Small Tailed Han Crossbred Sheep. Asian-Australas. J. Anim. Sci..

[10] Wang Z., Gerstein M., Snyder M. (2009). RNA-Seq: A Revolutionary Tool for Transcriptomics. Nat. Rev. Genet..

[11] Li G., Yang R., Lu X., Liu Y., He W., Li Y., Yu H., Qin L., Cao Y., Zhao Z. (2023). RNA-Seq Analysis Identifies Differentially Expressed Genes in the Longissimus Dorsi of Wagyu and Chinese Red Steppe Cattle. Int. J. Mol. Sci..

[12] Mortazavi A., Williams B.A., McCue K., Schaeffer L., Wold B. (2008). Mapping and Quantifying Mammalian Transcriptomes by RNA-Seq. Nat. Methods.

[13] Bai Y., Li H., Ji X., Wang W., Shao T., Chu Q., Yuan R., Wang Y., Liu L. (2026). Multi-Stage Transcriptomic Dissection Identifies Key Molecular Determinants of Chicken Follicular Development. Poult. Sci..

[14] Sun L., Bai M., Xiang L., Zhang G., Ma W., Jiang H. (2016). Comparative Transcriptome Profiling of Longissimus Muscle Tissues from Qianhua Mutton Merino and Small Tail Han Sheep. Sci. Rep..

[15] Wan S., Lou M., Zhang S., Li S., Ling Y. (2024). Transcriptome Analysis Revealed Differences in Gene Expression in Sheep Muscle Tissue at Different Developmental Stages. BMC Genom. Data.

[16] Song Y., Zhang Q., Shi J., Fu L., Cheng S. (2022). Screening of Genes Related to Growth, Development and Meat Quality of Sahan Crossbred F1 Sheep Based on RNA-Seq Technology. Front. Vet. Sci..

[17] Wang S., Jin J., Xu Z., Zuo B. (2019). Functions and Regulatory Mechanisms of lncRNAs in Skeletal Myogenesis, Muscle Disease and Meat Production. Cells.

[18] Kopp F., Mendell J.T. (2018). Functional Classification and Experimental Dissection of Long Noncoding RNAs. Cell.

[19] Cesana M., Cacchiarelli D., Legnini I., Santini T., Sthandier O., Chinappi M., Tramontano A., Bozzoni I. (2011). A Long Noncoding RNA Controls Muscle Differentiation by Functioning as a Competing Endogenous RNA. Cell.

[20] Gong C., Li Z., Ramanujan K., Clay I., Zhang Y., Lemire-Brachat S., Glass D.J. (2015). A Long Non-Coding RNA, LncMyoD, Regulates Skeletal Muscle Differentiation by Blocking IMP2-Mediated mRNA Translation. Dev. Cell.

[21] Yu X., Zhang Y., Li T., Ma Z., Jia H., Chen Q., Zhao Y., Zhai L., Zhong R., Li C. (2017). Long Non-Coding RNA Linc-RAM Enhances Myogenic Differentiation by Interacting with MyoD. Nat. Commun..

[22] Chao T., Ji Z., Hou L., Wang J., Zhang C., Wang G., Wang J. (2018). Sheep Skeletal Muscle Transcriptome Analysis Reveals Muscle Growth Regulatory lncRNAs. PeerJ.

[23] Lu J., Liu Y., Li H. (2024). Identification of Key lncRNAs and mRNAs in Muscle Development Pathways of Tan Sheep. Comp. Biochem. Physiol. Part D Genom. Proteom..

[24] Ren C., Deng M., Fan Y., Yang H., Zhang G., Feng X., Li F., Wang D., Wang F., Zhang Y. (2017). Genome-Wide Analysis Reveals Extensive Changes in LncRNAs during Skeletal Muscle Development in Hu Sheep. Genes.

[25] Liu W., Chen M., Liu Y., Li X., Li H., Wang J. (2025). Understanding lncRNAs: Key Regulators of Myogenesis and Lipogenesis in Farm Animals. Front. Vet. Sci..

[26] Chen Q., Bao J.-J., Zhang H.-C., Huang C., Zhao Q., Pu Y.-B., Jiang L., Hosseiny A., Ibrahim M., Hussain T. (2024). LncRNA GTL2 Regulates Myoblast Proliferation and Differentiation via the PKA-CREB Pathway in Duolang Sheep. Zool. Res..

[27] Yang F., Chi R., Zhang R., Liu K., Wang P., Di R., He X., Wang X., Liu Y., Chu M. (2025). A Novel ceRNA Axis LOC121818100/Novel-miR-400/SSRP1 Regulated Muscle Growth and Injury Repair in Sheep. J. Cachexia Sarcopenia Muscle.

[28] Yuan C., Zhang K., Yue Y., Guo T., Liu J., Niu C., Sun X., Feng R., Wang X., Yang B. (2020). Analysis of Dynamic and Widespread lncRNA and miRNA Expression in Fetal Sheep Skeletal Muscle. PeerJ.

[29] Li C.-Y., Li X., Liu Z., Ni W., Zhang X., Hazi W., Ma Q., Zhang Y., Cao Y., Qi J. (2019). Identification and Characterization of Long Non-Coding RNA in Prenatal and Postnatal Skeletal Muscle of Sheep. Genomics.

[30] Li Y., Wang Y., Yan M., Wu S., Liu M., Khan R. (2025). Screening and Functional Transformation Analysis of Genes Related to Skeletal Muscle Development in Supplemental-Fed Oula Sheep. Animals.

[31] Wei C., Wu M., Wang C., Liu R., Zhao H., Yang L., Liu J., Wang Y., Zhang S., Yuan Z. (2018). Long Noncoding RNA Lnc-SEMT Modulates IGF2 Expression by Sponging miR-125b to Promote Sheep Muscle Development and Growth. Cell Physiol. Biochem..

[32] Almada A.E., Horwitz N., Price F.D., Gonzalez A.E., Ko M., Bolukbasi O.V., Messemer K.A., Chen S., Sinha M., Rubin L.L. (2021). FOS Licenses Early Events in Stem Cell Activation Driving Skeletal Muscle Regeneration. Cell Rep..

[33] Zhang X., Liu C., Kong Y., Li F., Yue X. (2022). Effects of Intramuscular Fat on Meat Quality and Its Regulation Mechanism in Tan Sheep. Front. Nutr..

