# Supplementary material for: Integrated lncRNA and mRNA Transcriptome Analysis Reveals Candidate Regulatory Networks Associated with Postnatal Longissimus Dorsi Muscle Development in Hanper Sheep
Source: Biology (Basel). 2026 Sep 23;15(19):1694. doi: 10.3390/biology15191694 (PMC13663505; doi:10.3390/biology15191694)
Supplement: Supplementary file 1 [file biology-15-01694-s001.zip › Figure S1 and Table S1.pdf]

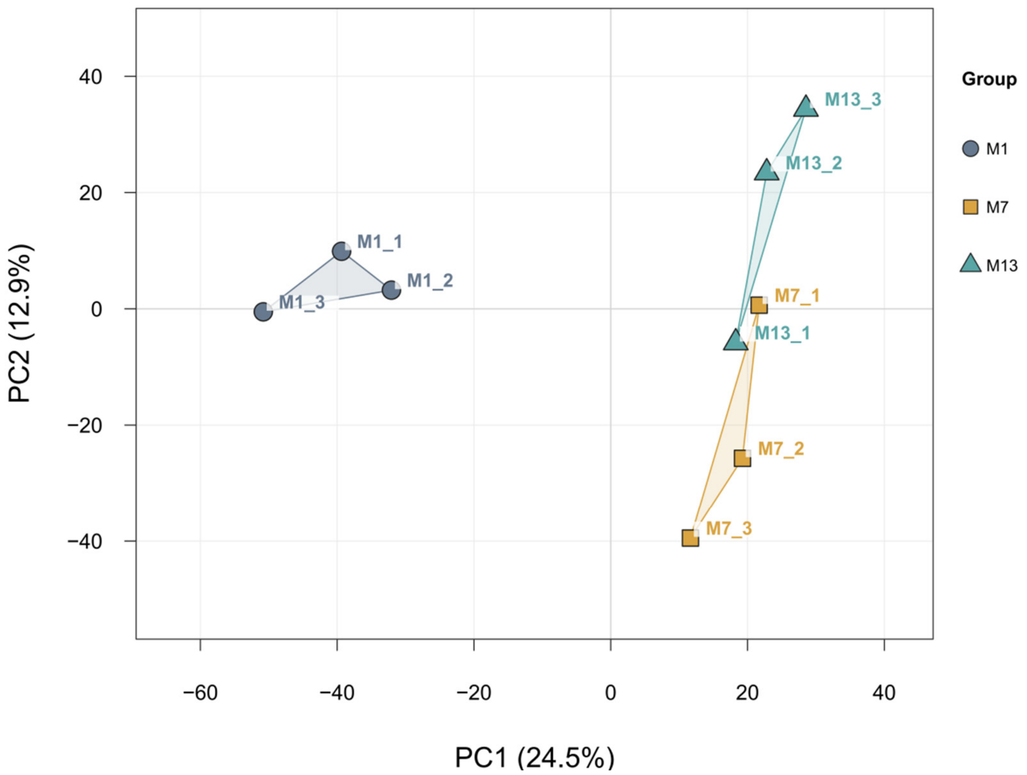

Figure S1. Principal component analysis (PCA) of RNA-seq samples from Hanper sheep longissimus dorsi muscle.

PCA was performed based on the log2(FPKM + 1)-transformed expression matrix of expressed mRNAs and lncRNAs. Each point represents one biological replicate, and different colors and shapes indicate the three developmental stages, including M1, M7, and M13. PC1 and PC2 explained 24.5% and 12.9% of the total variance, respectively.

Table S1. Summary of RNA-seq data quality and mapping statistics.

Sample	Raw reads	Clean reads	Clean bases	Error rate (%)	Q20 (%)	Q30 (%)	GC content (%)	Total mapped (%)	Uniquely mapped (%)	Multiple mapped (%)	Properly paired (%)
M1_1	111,672,488	106,148,502	15.92G	0.01	97.62	93.83	54.06	93.04	75.57	17.47	71.82
M1_2	118,212,162	114,941,852	17.24G	0.01	97.97	94.72	54.31	93.31	72.00	21.31	68.93
M1_3	104,711,028	101,057,144	15.16G	0.01	97.94	94.46	54.34	92.21	73.09	19.12	69.78
M7_1	109,390,682	105,241,778	15.79G	0.01	97.90	94.34	55.99	89.66	69.33	20.33	65.67
M7_2	116,141,152	113,110,994	16.97G	0.01	97.84	94.41	55.45	88.32	73.46	14.86	69.52
M7_3	112,985,388	110,764,330	16.61G	0.01	97.69	94.13	53.76	89.35	78.01	11.34	73.50
M13_1	106,868,610	104,489,324	15.67G	0.01	97.66	94.08	53.66	88.20	77.65	10.55	73.07
M13_2	109,269,680	106,393,160	15.96G	0.01	97.70	94.13	55.21	86.22	74.74	11.48	70.45
M13_3	94,633,718	91,319,600	13.70G	0.01	97.45	93.31	55.10	87.25	74.82	12.42	70.97
